# Dr. Harrison on Sterility

**Published:** 1831-04-01

**Authors:** 


### DR. HARRISON ON STERILITY.

THIS is as regular a puff as ever emanated from St. John Long or Dr. Eady. The SPINE-TINKERING trade has probably declined of late in Holles-street; and a new tub is now thrown out to the whale—in the shape of a bubble-hypothesis that sterility depends on crooked spines—and as Dr. Harrison proclaims himself the best spine-doctor in Modern Babylon, the inference is natural enough, that the impotent and barren of both sexes will fly to Holles-street, to have their backs thumbed and their families increased :—Listen to the boastings of the spine-doctor. "*My* successful exertions in removing derangements of the spinal column, and *ability* to rectify its numerous deflections, have already led to a better knowledge of the *peculiar* functions of the spinal *nerves*, both in their healthy and diseased condition." Did ever regular physician put forth such a piece of puffery in this world! Then comes a leash of *anonymous* cases, where females ceased to bear children while their health was bad—had their spines thumbed by the Doctor for real or supposed deformities—got into good health—and then became pregnant—ergo, quoth the Doctor, it was the spinal *deflection* that caused sterility for a time; and my thumbs, by putting the vertebræ into their proper places, restored the uterine system to its proper vigour—Q. E. D.!

There is not a more crafty old fox within this wide metropolis than the spine-doctor of Holles-street!

